# Usefulness of combined NGS and QF‐PCR analysis for product of conception karyotyping

**DOI:** 10.1002/rmb2.12449

**Published:** 2022-02-27

**Authors:** Takema Kato, Shunsuke Miyai, Hideki Suzuki, Yuuri Murase, Shiyo Ota, Hiroko Yamauchi, Michiko Ammae, Tatsuya Nakano, Yoshiharu Nakaoka, Tomoko Inoue, Yoshiharu Morimoto, Aisaku Fukuda, Takafumi Utsunomiya, Haruki Nishizawa, Hiroki Kurahashi

**Affiliations:** ^1^ Division of Molecular Genetics Institute for Comprehensive Medical Science Fujita Health University Aichi Japan; ^2^ OVUS Inc. Aichi Japan; ^3^ IVF Namba Clinic Osaka Japan; ^4^ HORAC Grand Front Osaka Clinic Osaka Japan; ^5^ IVF Osaka Clinic Osaka Japan; ^6^ St. Luke Clinic Oita Japan; ^7^ Department of Obstetrics and Gynecology Fujita Health University School of Medicine Aichi Japan

**Keywords:** assisted reproductive technology, karyotype, next‐generation sequencing, product of conception, triploidy

## Abstract

**Purpose:**

Since chromosomal abnormalities can be detected in more than half of miscarriages, cytogenetic testing of the product of conception (POC) can provide important information when preparing for a subsequent pregnancy. Conventional karyotyping is the common diagnostic method for a POC but can be problematic due to the need for cell culture.

**Methods:**

We here conducted shallow whole‐genome sequencing (sWGS) using next‐generation sequencing (NGS) for alternative POC cytogenomic analysis. Since female euploidy samples can include 69,XXX triploidy, additional QF‐PCR was performed in these cases.

**Results:**

We here analyzed POC samples from miscarriages in 300 assisted reproductive technology (ART) pregnancies and detected chromosomal abnormalities in 201 instances (67.0%). Autosomal aneuploidy (151 cases, 50.3%) was the most frequent abnormality, consistent with prior conventional karyotyping data. Mosaic aneuploidy was detected in seven cases (2.0%). Notably, the frequency of triploidy was 2.3%, 10‐fold lower than the reported frequency in non‐ART pregnancies. Structural rearrangements were identified in nine samples (3%), but there was no case of segmental mosaicism.

**Conclusions:**

These data suggest that NGS‐based sWGS, with the aid of QF‐PCR, is a viable alternative karyotyping procedure that does not require cell culture. This method could also assist with genetic counseling for couples who undergoes embryo selection based on PGT‐A data.

## Graphical abstract

sWGS by NGS combined with QF‐PCR is the optimal diagnostic method for cytogenetic testing of POC samples.

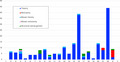

## 1 INTRODUCTION

10–15% of clinically recognized pregnancies result in miscarriage, among which there is a further recurrent pregnancy loss rate of 1%.^1^ Many factors are known to cause miscarriages, but more than 50% are due to chromosomal abnormalities.^2^ Chromosome testing of a product of conception (POC) reveals not only the cause of the miscarriage but also provides important clinical information to assist couples preparing for a subsequent pregnancy.^3^ The most common chromosomal abnormality to cause a miscarriage is an autosomal trisomy, followed by monosomy X and then polyploidy.^2^, ^4^ Hence, conventional G‐banding has long been used to screen chromosomal abnormalities in POC samples. However, despite the importance of chromosome testing of POC samples, only 8% of miscarriages have actually been tested.^5^ The G‐banding of POC samples has practical limitations related to the need for cell culturing which can often fail due to fetal demise or macerated tissue, the preferential growth of maternal decidua cells, and the emergence of artifacts.^6^, ^7^, ^8^ Recently, SNP microarray, next‐generation sequencing (NGS), quantitative fluorescence PCR (QF‐PCR), and multiplex ligation‐dependent probe amplification (MLPA), none of which require cell culture, have been reported as alternative approaches to the cytogenomic analysis of a POC.^9^, ^10^, ^11^, ^12^, ^13^, ^34^

NGS is a powerful tool that can allow both qualitative and quantitative analyses to be performed simultaneously. Currently, NGS‐based chromosome analysis is mainly used in the pre‐implantation genetic testing for aneuploidy and structural rearrangements (PGT‐A and PGT‐SR), using the amplified products of the whole genome from the biopsied trophectoderm cells of a 5‐day embryo.^14^, ^15^ Chromosomal copy number analysis via NGS does not require a massive amount of data since it is determined using shallow whole‐genome sequencing (sWGS). In addition, these data can be obtained within 24 hours. Moreover, sWGS by NGS enables the simultaneous analysis of a large number of samples, thus reducing the cost per sample. A number of validation experiments for these methods, such as mosaic sensitivity and resolution by NGS‐based sWGS, have been described.^15^, ^16^, ^17^, ^18^, ^19^, ^20^ On the other hand, this method is limited in its ability to detect polyploidy, which is a common genetic cause of miscarriage. Whereas triploidy containing a Y chromosome can be detected by calculating the sex chromosome ratio, polyploidy without a Y chromosome, such as 69,XXX and 92,XXXX, is very difficult to distinguish from 46,XX using NGS‐based sWGS.

In our present study, we utilized NGS‐based sWGS for the chromosome analysis of POCs from ART pregnancies. We characterized the chromosomal abnormalities in the POC, and the usefulness of NGS‐based sWGS for this screening. Further, to overcome limitations with this approach in the detection of triploidy, we evaluated the usefulness of including QF‐PCR analysis in a subset of samples.

## 2 MATERIALS AND METHODS

### 2.1 Samples

After obtaining informed consent, chorionic tissue sampling was conducted after first‐trimester miscarriages following ART. Under an anatomic microscope, chorionic tissues were washed with phosphate buffer solution to remove coagulated blood clots. After the maternal decidua was removed, chorionic villi were selected by trained laboratory staff. A total of 300 samples were received for chromosome analysis. Oocyte insemination was performed by either conventional in vitro fertilization (n = 103) or by intracytoplasmic sperm injection (ICSI) (*n* = 155). The mean age of the pregnant women was 37.3 years (range, 25–47 years). Fetal heartbeat was confirmed in 175 cases, and the mean gestational age at miscarriage was 8.7 weeks (range, 6.4–10.9 weeks).

### 2.2 sWGS by next‐generation sequencing

The karyotypes in the ART pregnancies were determined by sWGS by NGS. Genomic DNA was extracted from chorionic villi or fetus tissue using Gentra Puregene Tissue Kit (Qiagen Inc., Valencia, CA), in accordance with the manufacturer's protocol. Genomic DNA samples were then diluted to 1ng/μl for whole‐genome amplification (WGA). Subsequently, WGA was performed using the SurePlex WGA Kit (Illumina, San Diego, CA) in accordance with the manufacturer's instructions. Nextera libraries were prepared from the amplified DNA and subsequently sequenced with a VeriSeq PGS assay system by MiSeq (Illumina). The sequencing data were analyzed using BlueFuse Multi analysis software v4.5.

### 2.3 QF‐PCR

QF‐PCR analysis was performed on 53 cases with 46,XX or mosaic aneuploidy karyotypes to confirm the possible presence of triploidy. An Aneufast QF‐PCR kit was used for this analysis, according to the manufacturer's protocol (Genomed Diagnostics AG, Switzerland). The kit contains multiplex marker sets of short tandem repeats (STRs) that can be used for amplification of selected microsatellites on chromosomes 13, 18, 21, and X. All PCR products were genotyped on an ABI3130 Genetic Analyzer using GeneMapper analysis software (Thermo Fisher Scientific).

## 3 RESULTS

**FIGURE 1:**
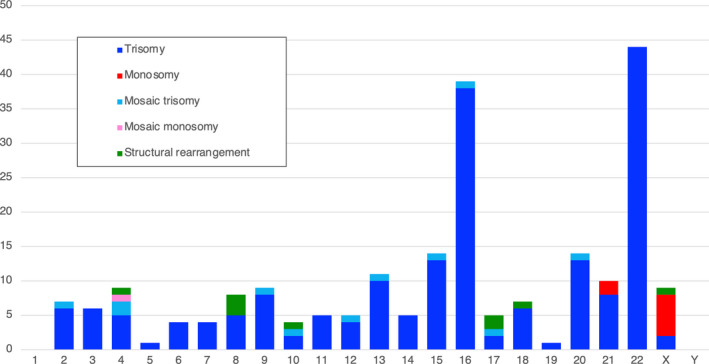
Description of the detected chromosomal abnormalities in the POC samples. Bar graphs indicating the frequencies of abnormal chromosomes in the indicated chromosomal abnormalities detected by sWGS using NGS. Blue bars indicate trisomy, while red bars indicate monosomy. Regarding mosaicism, the light blue and pink bars indicate trisomy and monosomy, respectively. Green bars indicate structural rearrangements. SR indicates structural rearrangement

We performed NGS‐based sWGS for 300 POC samples obtained from an ART miscarriage. We detected chromosomal abnormalities in 197 cases (65.7%; Table 1). Autosomal aneuploidy (50.3%) was the most frequently observed abnormality. As was expected, trisomy 16 and 22 were the first and the second most frequent anomalies, while trisomy 15 was the third most commonly observed (Figure 1). In contrast, autosomal monosomy was only found on chromosome 21 in this series. Twenty samples showed multiple aneuploidies involving two or more chromosomes (6.7%). With regard to sex chromosome aneuploidies, monosomy X was observed in 6 samples (2%), which could be considered unexpectedly low. Aneuploidy involving an increased number of sex chromosomes was observed in two of the cases with multiple aneuploidies.

**TABLE 1:** Types of abnormal karyotypes in the analyzed POC samples

Karyotype	NGS		NGS +QF‐PCR	
Normal karyotype	103	34.3%	99	33.0%
Autosomal aneuploidy	151	50.3%	151	50.3%
Multiple aneuploidy	20	6.7%	20	6.7%
Autosomal mosaic aneuploidy	7	2.3%	6	2.0%
Sex chromosome aneuploidy	6	2.0%	6	2.0%
Polyploidy	4	1.3%	7	2.3%
Structural rearrangements	9	3.0%	9	3.0%
Whole chromosome uniparental isodisomy	0	0.0%	2	0.7%
Total	300		300	

With the NGS‐based sWGS method, we could identify triploidy from an abnormal X/Y ratio in cases containing both X and Y chromosomes. Triploidy was detected in this way in four XXY cases (1.3%), which was a considerably low frequency. Mosaicism was detected in seven cases (2.3%), all of which were instances of mosaic aneuploidy without any predominant chromosome (2 cases of trisomy 4, 1 case of monosomy 4, and 1 case each of trisomy 10, 16, 17, and X). Structural rearrangements were identified in 9 samples (3%), and there was no identified case of segmental mosaicism. The data are summarized in Table 1 (*left*).

We observed in our present sample series that the male/female ratio was slightly biased toward females among the allegedly normal cases (i.e., male: female, 46:57), and speculated that this subgroup may have included some 69,XXX karyotypes. We performed QF‐PCR to distinguish between 69,XXX and 46,XX samples. QF‐PCR can also distinguish true mosaicism from maternal contamination. We performed this testing on all the 57 allegedly normal female cases as well as 7 cases with mosaicism. The results indicated that 2 of these 57 samples had a 69,XXX karyotype. In addition, one of the mosaic cases that were previously diagnosed as mosaic X monosomy was found to be triploid. Even after recalculating that number, the total number of triploidy cases was 7 (2.3%), a substantially lower frequency than expected. In addition, two 46,XX samples were found to be whole chromosome uniparental disomy cases. The results revised by QF‐PCR are also summarized in Table 1 (*right*). The final male/female ratio in our current series was still slightly biased toward female (46:53).

## 4 DISCUSSION

We have here demonstrated the utility of the sWGS via the NGS method for the cytogenetic testing of a miscarried fetus. Using this approach to analyze POCs from 300 ART miscarriages revealed chromosome abnormalities in 197 (65.7%) cases, which increased to 201 (67.0%) of the samples when we combined QF‐PCR analysis. Importantly, these are similar values to those previously published in prior studies using more conventional culture‐based karyotyping methods.^2^ Additionally, the frequency of each chromosomal abnormality in our current sample series was also similar to that reported in these studies. Trisomy 16, 22, and 15 have been the most frequently observed of the autosomal aneuploidies in POC,^4^, ^9^, ^21^, ^22^, ^23^, ^24^, ^25^ whereas trisomies 13, 18, and 21 are the commonly found neonatal chromosomal abnormalities. Small chromosomes are predisposed to meiotic error since they tend to carry distally placed chiasmata that might be susceptible to breakage during the long arrest of meiosis I.^26^ Another factor is the timing of the selection of trisomy cells or trisomy fetuses. Trisomies 16, 22, and 15 can be subject to weaker adverse selection than the other autosomal trisomies involving the larger chromosomes and can survive until the later stages of the first trimester. In contrast, chromosomes 13, 18, and 21 are the most gene‐poor chromosomes, resulting in the weakest adverse selection. On the contrary, trisomy of chromosomes 1, 5, and 19 are rare, suggesting that they might contain genes that are highly important during early embryogenesis. Since autosomal monosomies are not yet selected at the blastocyst stage used in the PGT‐A chromosome diagnosis, they are found at a similar frequency to autosomal trisomies.^27^ It is thought that many autosomal monosomies undergo selection at the early stage of the first trimester.^21^

Notably with regard to our present findings, the frequency of triploidy was only 7/300 (2.3%), which is considerably lower than the previously reported frequencies in POCs. However, our current study sample source, POCs from ART pregnancies, may have been the important contributor to this result. Since most ART pregnancies are achieved by ICSI, instances of diandric triploidy, dispermy or diploid sperm may have been avoided in our present series via microscopic examination and manipulation (only one in seven POCs with triploidy was by ICSI). Further to this, a microscopic confirmation of fertilization can help to exclude 3PN embryos for transplantation, also leading to a low frequency of triploidies in ART pregnancies.^28^ A similar tendency toward a lower frequency of polyploidies in ART pregnancies compared with natural pregnancies has also been observed in other previous reports.^23^, ^29^, ^30^ These findings suggest, importantly, that although the karyotyping system in PGT‐A cannot distinguish 69,XXX from 46,XX, the detection of triploidies is not necessarily important for PGT‐A since all are ART pregnancies.

Unexpectedly from our current analyses, the frequency of monosomy X was low (6 cases, 2.0%) compared with that observed in natural pregnancies in previous reports.^25^, ^26^ Monosomy X is known to originate from mitotic errors in cleavage stage embryos, suggesting that most of the cases will manifest somatic mosaicism.^31^, ^32^ The samples we collected from chorionic villi without culturing were mainly trophoblasts, whereas cells analyzed using a standard method are mainly mesenchymal cells of fetal origin. The differences between the developmental stages in these two scenarios may have an impact on the level of sex chromosome loss, leading to differences in the detection rate of monosomy X. A more thorough analysis of low‐level mosaicism in monosomy X would help to validate this hypothesis.

The detection of subtle structural rearrangements requires high‐resolution chromosome analysis. Notably, however, sWGS via the NGS method, which has been used in PGT‐A/SR, is known to have a similar resolution to that of G‐banding which still cannot detect subtle structural rearrangements of less than 10 Mb.^16^, ^17^ Moreover, the frequency of structural rearrangements in our POC samples was 3.0%, which is comparable to that obtained previously in POCs using higher resolution cytogenetic microarrays.^4^, ^23^ This suggests that high‐resolution chromosome analysis may be redundant for POC analysis and that sWGS is sufficient to detect the cytogenetic abnormalities that contribute to miscarriage. We also did not observe any segmental mosaicism in our current POC series. In PGT‐A, a substantial subset of embryos shows segmental mosaicism. Despite the typically favorable outcomes of these anomalies, genetic counseling is still required.^33^ Our current data suggest however that most of these cases might be artifacts generated by whole‐genome amplification using genomic DNA derived from a small number of cells.

In summary, sWGS by NGS has a resolution comparable to G‐banding, although it cannot be used to observe balanced chromosomal abnormalities. This cytogenetic methodology that does not require the use of cell culture has many advantages, such as avoiding the effects of maternal cell contamination. Since POC samples can be stored temporarily in a freezer, it is also possible to test for miscarriages that occurred outside the hospital with this new approach. Further to this, the medical staff can offer cytogenetic testing at a later and more appropriate time after the couple have recovered from the grief caused by the miscarriage. Importantly also, we contend that its low cost and short turnaround time (<24 hours) makes sWGS by NGS a better option for the cytogenetic analysis of POC samples. This technique may therefore be useful in the future for the cytogenetic testing of chorionic villi samples or amniocenteses, or as a confirmation test after NIPT.

## CONFLICT OF INTEREST

Takema Kato held a cross appointment as Assistant Professor at the Fujita Health University and in the OVUS Inc. Shunsuke Miyai, Hideki Suzuki and Yuuri Murase are full‐time employees of OVUS Inc. Shiyo Ota, Hiroko Yamauchi, Michiko Ammae, and Tatsuya Nakano are full‐time employees of IVF namba clinic. Yoshiharu Nakaoka is the director of IVF Namba clinic. Tomoko Inoue is a full‐time employee of HORAC Grandfront Osaka Clinic. Yoshiharu Morimoto is the director of HORAC Grandfront Osaka Clinic. Aisaku Fukuda is the director of IVF Osaka Clinic. Takafumi Utsunomiya is the director of St. Luke Clinic. Haruki Nishizawa declare that they have no conflict of interest. Hiroki Kurahashi is the director of OVUS Inc.

## HUMAN RIGHTS STATEMENTS AND INFORMED CONSENT

All samples were used after receiving written informed consent from the participating patients and family members. The study was approved by the Ethical Review Committee for Human Genome Studies at Fujita Health University and was conducted in compliance with the Declaration of Helsinki.

## ANIMAL STUDIES

This article does not contain any studies with animal subjects performed by any of the authors.

## APPROVAL BY ETHICS COMMITTEE

The study was approved by the Ethics Review Committee for Human Genome Studies at Fujita Health University.

## CLINICAL TRIAL REGISTRY SUBSECTIONS

This article does not contain the data of the clinical trial registry.
